# Activation of calpain by renin-angiotensin system in pleural mesothelial cells mediates tuberculous pleural fibrosis

**DOI:** 10.1152/ajplung.00348.2015

**Published:** 2016-06-03

**Authors:** Jie Yang, Fei Xiang, Peng-Cheng Cai, Yu-Zhi Lu, Xiao-Xiao Xu, Fan Yu, Feng-Zhi Li, Peter A. Greer, Huan-Zhong Shi, Qiong Zhou, Jian-Bao Xin, Hong Ye, Yunchao Su, Wan-Li Ma

**Affiliations:** ^1^Department of Respiratory and Critical Care Medicine, Union Hospital, Tongji Medical College, Huazhong University of Science and Technology, Wuhan, Hubei, China;; ^2^Key Laboratory of Respiratory Diseases, Ministry of Health of China, Wuhan, Hubei, China;; ^3^Department of Clinical Laboratory, Union Hospital, Tongji Medical College, Huazhong University of Science and Technology, Wuhan, Hubei, China;; ^4^Queen's University Cancer Research Institute, Kingston, Ontario, Canada;; ^5^Department of Respiratory and Critical Care Medicine, Beijing Chaoyang Hospital, Capital Medical University, Beijing, China;; ^6^Department of Pathophysiology, Tongji Medical College, Huazhong University of Science and Technology, Wuhan, Hubei, China; and; ^7^Department of Pharmacology and Toxicology, Medical College of Georgia, Georgia Regents University, Augusta, Georgia

**Keywords:** calpain, pleural mesothelial cell, fibrosis, tuberculosis, pleural effusion

## Abstract

Pleural fibrosis is defined as an excessive deposition of extracellular matrix (ECM) components that results in destruction of the normal pleural tissue architecture. It can result from diverse inflammatory conditions, especially tuberculous pleurisy. Pleural mesothelial cells (PMCs) play a pivotal role in pleural fibrosis. Calpain is a family of calcium-dependent endopeptidases, which plays an important role in ECM remodeling. However, the role of calpain in pleural fibrosis remains unknown. In the present study, we found that tuberculous pleural effusion (TPE) induced calpain activation in PMCs and that inhibition of calpain prevented TPE-induced collagen-I synthesis and cell proliferation of PMCs. Moreover, our data revealed that the levels of angiotensin (ANG)-converting enzyme (ACE) were significantly higher in pleural fluid of patients with TPE than those with malignant pleural effusion, and ACE-ANG II in TPE resulted in activation of calpain and subsequent triggering of the phosphatidylinositol 3-kinase (PI3K)/Akt/NF-κB signaling pathway in PMCs. Finally, calpain activation in PMCs and collagen depositions were confirmed in pleural biopsy specimens from patients with tuberculous pleurisy. Together, these studies demonstrated that calpain is activated by renin-angiotensin system in pleural fibrosis and mediates TPE-induced collagen-I synthesis and proliferation of PMCs via the PI3K/Akt/NF-κB signaling pathway. Calpain in PMCs might be a novel target for intervention in tuberculous pleural fibrosis.

pleural fibrosis is defined as an excessive deposition of extracellular matrix that results in destruction of the normal pleural tissue architecture and compromised function. The disease is caused by diverse inflammatory conditions, its main causes are tuberculous pleurisy, asbestos injury, and rheumatoid pleurisy (19). Tuberculous pleurisy is the most frequent extrapulmonary manifestation of tuberculosis (25). When tuberculous pleural effusions (TPE) resolve, pleural thickening occurs in about half of patients (11). However, a complete understanding of the pathogenesis of pleural fibrosis remains unknown. Moreover, pleural fibrosis is irreversible. In some severe cases, the progression of pleural fibrosis leads to lung entrapment, resulting in dyspnea and respiratory failure. Pleural decortication is eventually the only treatment option.

Pleural fibrosis typically occurs following severe pleural inflammation, which is associated with exudative pleural effusion. Both TPE and malignant pleural effusion (MPE) are exudative pleural effusion. However, pleural fibrosis seldom occurs following MPE. Transforming growth factor-β_1_ (TGF-β_1_) is a key factor in fibrosis; however, the levels of TGF-β_1_ are similar between TPE and MPE (5, 12). Thus other reactive substances should be sought for the mechanism of pleural fibrosis.

Pleural mesothelial cells (PMCs) play a pivotal role in pleural fibrosis. Although the mechanism of pleural fibrosis is not fully understood, the response of the PMCs and their ability to regenerate are important determinants of pleural fibrosis (19). PMCs in both visceral and parietal pleura are similarly sensitive and responsive to various stimuli and are easily injured. Berg et al. (1) reported that PMCs are more susceptible to H_2_O_2_- and SiO_2_-induced oxidative stress and damage than lung epithelial cells. Moreover, reactive PMCs also take part in pleural inflammation by upregulating vasoactive substances and enzymes in the oxidative pathway (19, 32). PMCs proliferate and excrete plasminogen activator inhibitor-1 (PAI-1) (8) and extracellular matrix to repair the pleural injury. Proliferation of PMCs and overdeposition of extracellular matrix in response to injury result in pleural fibrosis. The phosphatidylinositol 3-kinase (PI3K)/Akt/NF-κB pathway has been reported to be involved in cell proliferation and extracellular matrix regulation of PMCs (31).

Our recent study revealed that calpain, a calcium-dependent nonlysosomal neutral cysteine endopeptidase, plays a pivotal role in collagen synthesis and cell proliferation in pulmonary vascular remodeling (22). Tabata et al. (26) reported that a calpain inhibitor prevents bleomycin-induced pulmonary fibrosis in mice. However, it is not known how calpain is activated.

The renin-angiotensin (ANG) system (RAS) is a key regulator of blood pressure and fluid homeostasis. ANG II is produced by the action of renin on angiotensinogen to form ANG I, which is further cleaved by angiotensin-converting enzyme (ACE) to form ANG II. ACE and ANG II have been proposed to be involved in fibrotic diseases, such as cardiovascular fibrosis (30) and pulmonary fibrosis (29). ANG II has been reported to activate calpain in vascular smooth muscle cells (24). Therefore, we hypothesize that ANG II-induced calpain activation plays an important role in collagen synthesis and cell proliferation of PMCs in pleural fibrosis. In the current study, we used a cell model of PMCs exposed to TPE, MPE, or ANG II and clinical samples to confirm our hypothesis. The observations indicate that calpain in PMCs might be a novel target for intervention in tuberculous pleural fibrosis.

## MATERIALS AND METHODS

### 

#### Human subjects.

The study protocol was approved by the Institutional Review Board of the Tongji Medical College, Huazhong University of Science and Technology. Informed consent was obtained from all subjects. There were 56 patients with TPE, and 32 patients with MPE. The criteria for TPE were the identification of *Mycobacterium tuberculosis* in pleural fluid or the demonstration of granulomatous pleurisy in closed pleural biopsy specimen in the absence of any evidence of other granulomatous diseases. The criteria for MPE were the demonstration of cancerous cells in pleural fluid or in closed pleural biopsy specimen. At the time of sample collection, none of the patients had received any antituberculosis therapy, anticancer therapy, corticosteroids, or other nonsteroid anti-inflammatory drugs.

#### Pleural effusion samples collection and processing.

Five-hundred to one-thousand milliliters of TPE or MPE samples from each patient were collected in heparin-treated tubes, through a standard thoracocentesis technique within 24 h after hospitalization. Twenty milliliters of blood were drawn simultaneously. TPE or MPE specimens were immersed in ice immediately and then centrifuged at 1,200 *g* for 5 min. Supernatants were aliquoted and stored at −80°C for experiments.

#### Reagents.

ANG II, the calpain inhibitor MDL28170, the PI3K inhibitor LY294002, and fluorogenic peptide Suc-Leu-Leu-Val-Tyr-AMC were obtained from Calbiochem (La Jolla, CA). Anti-collagen-I antibody was obtained from Novus Biologicals (Littleton, CO). Antibodies against phospho-Akt (p-Akt) and total-Akt (t-Akt) were purchased from Cell Signaling Technology (Danvers, MA). NF-κB p65 was obtained from Santa Cruz Biotechnology (Dallas, TX). Antibodies against IκB-α and GAPDH were obtained from Epitomics (Burlingame, CA). Anti-calretinin antibody was purchased from BD Transduction (San Jose, CA). Bromodeoxyuridine (BrdU) kit was obtained from Roche (Mannheim, Germany). Enzyme-linked immunosorbent assay (ELISA) kits of ACE were from R&D Systems (Minneapolis, MN). The type 1 ANG II receptor antagonist losartan was obtained from Sigma-Aldrich (St. Louis, MO). Antibody specific for the calpain-mediated cleavage of spectrin (SBDP) was provided by Dr. Kevin K. W. Wang.

#### PMCs culture and treatment.

The human PMC line (MeT-5A) was purchased from American Type Culture Collection (ATCC, Manassas, VA). The PMCs were cultured in Roswell Park Memorial Institute (RPMI) 1640 medium (Hyclone, Logan, UT) supplemented with 20% fetal calf serum and 5% CO_2_-95% air at 37°C. The cells were subcultured 1:3 when cells grew to confluence, and culture medium was changed every 2 days. Cells equilibrated in serum-free medium overnight were used for all experiments as in a study by Tucker et al. (28). Then, the cells were treated by using TPE, serum from the same patient with TPE (STB), MPE (final concentration of TPE, MPE, or STB in the serum-free medium was 2%), with or without inhibitors, for the time periods indicated.

#### Isolation and primary culture of rat PMCs.

Primary PMCs were isolated from rat pleura with pronase E digestion. In brief, the rat was anesthetized by intraperitoneal injection with 7% chloral hydrate (5 μl/g body wt) and then 5 ml 1% pronase E in RPMI-1640 were injected into thoracic cavity. The rat was then killed by using carbon dioxide. The whole thorax was isolated by cutting the lumbar vertebrae, cervical vertebrae, and connected tissues under sterile conditions, and the pleura was digested by pronase E at 4°C overnight. PMCs were harvested from thoracic cavity and centrifuged at 1,000 rpm for 5 min. The spun down cells were resuspended with epithelial cell medium (Cell Biologics, Chicago, IL) and cultured in a 5% CO_2_ incubator at 37°C.

#### Western blot analysis.

Intracellular protein levels were measured by using Western blot analysis as described in our previous study (7). The cell lysates (10–20 μg of protein) were denatured and electrophoresed on SDS-PAGE gels. Separated proteins were electro-transferred to nitrocellulose membranes. The primary antibodies were against collagen-I (dilution 1:1,000), p-Akt (dilution 1:1,000), t-Akt (dilution 1:1,000), IκB-α (dilution 1:5,000), or GAPDH (dilution 1:8,000). At last, films were developed using Kodak Medical X-ray processor 102 (Kodak, Rochester, NY) to visualize reactive proteins followed by densitometric quantification using Image-Pro Plus software (Media Cybernetics, Rockville, MD).

#### Measurement of calpain activity in PMCs.

Calpain activity in PMCs was measured using the fluorogenic peptide Suc-Leu-Leu-Val-Tyr-AMC as a substrate following the procedure described previously (22). Briefly, the cells were cultured in 24-well plates. After being washed twice with phosphate-buffered saline (PBS), Suc-Leu-Leu-Val-Tyr-AMC was added to a final concentration of 80 μM in growth factor-free medium in the presence and absence of the calpain inhibitor MDL28170. Immediately after addition of Suc-Leu-Leu-Val-Tyr-AMC, fluorescence was recorded at 1-min intervals for 30 min at excitation of 360 nm and emission of 460 nm using a Synergy 2 Multi-Mode microplate reader (Biotek Instruments, Winooski, VT). Calpain activity was expressed by the fluorescence unit in the absence of MDL28170 subtracted from that in the presence of MDL28170.

#### Cell proliferation assay.

Proliferation of PMCs was assayed using a kit from Roche (Indianapolis, IN) that monitors the incorporation of BrdU into newly synthesized DNA. The BrdU was detected using anti-BrdU-peroxidase conjugated in accordance with the manufacturer's instructions. After reactions were stopped, the absorbance at 450 nm was measured using a Synergy 2 Multi-Mode microplate reader (Biotek Instruments, Winooski, VT).

#### Immunofluorescence staining of PMC.

To determine the intracellular localization and changes of collagen-I, PMCs were incubated with TPE for 24 h. Then, the cells were stained using rabbit polyclonal antibody against collagen-I and mouse monoclonal antibody against calretinin at 4°C overnight and then incubated with FITC-conjugated goat anti-rabbit and Cy3-conjugated goat anti-mouse secondary antibody at room temperature for 60 min. The nuclei were stained for DAPI for 10 min in dark. The fluorescence-labeled cells were examined using a fluorescent microscope (Olympus FV500, Olympus, Tokyo, Japan).

#### NF-κB activity analysis.

An ELISA-based analysis was used to measure the NF-κB activity as described previously (20). In brief, the cells were rinsed twice with cold PBS, detached with trypsin, and centrifuged for 10 min at 1,000 rpm. The pellets were then resuspended in 100 μl lysis buffer (20 mmol/l HEPES, pH 7.5, 0.35 mol/l NaCl, 20% glycerol, 1% NP-40, 1 mmol/l MgCl_2_, 0.5 mmol/l EDTA, and 0.1 mmol/l EGTA) containing a protease inhibitor cocktail. After incubation on ice for 10 min, the lysates were centrifuged for 20 min at 14,000 rpm. The supernatants constitute the total protein extract. After being quantified with BCA reagent, the cell extracts were kept frozen at −80°C until NF-κB activity was measured. Cell extracts were incubated in a 96-well plate coated with the oligonucleotide containing the NF-κB consensus-binding site (5′-GGGACTTTCC-3′). Activated transcription factors from extracts specifically bound to the respective immobilized oligonucleotide. NF-κB activity was then detected with the primary antibody to NF-κB p65 and secondary antibody conjugated to horseradish peroxidase. NF-κB activity was finally determined as absorbance value that was measured by a microplate reader at a wavelength of 450 nm.

#### Measurement of ACE protein level in pleural effusion.

The protein levels of ACE in TPE and MPE were measured using an ELISA kit from R&D Systems, according to the manufacturer's instructions.

#### Analysis of calpain activation in human pleura.

Human pleura slides were obtained from pleura biopsies of patients with TPE and MPE. To measure the level of calpain activation in pleura, spectrin breakdown product (SBDP) was measured using an antibody that specifically recognizes SBDP as described previously (10, 15). Spectrin contains a specific calpain cleavage site, releasing SBDP. This method has been widely used to detect calpain activation in vivo (3, 10, 15). The slides were double-labeled for SBDP and calretinin (specific marker of PMCs) and counterstained using DAPI. The slides were mounted with anti-fade reagent and examined using a fluorescent microscope (Olympus FV500; Olympus, Tokyo, Japan). The yellow color indicates colocalization of SBDP and calretinin.

#### Statistical analysis.

In each experiment, control and experimental cells were matched for cell line, age, seeding density, number of passages, and number of days postconfluence to avoid variation in tissue culture factors that can influence the measurements of proteins. Results are shown as the mean ± SE. Differences between groups were analyzed using unpaired *t*-tests or two-way ANOVA. *P* < 0.05 was considered to be statistically significant.

## RESULTS

### 

#### TPE induced an increase in collagen-I synthesis and proliferation of PMCs by activation of calpain.

**Fig. 1.**
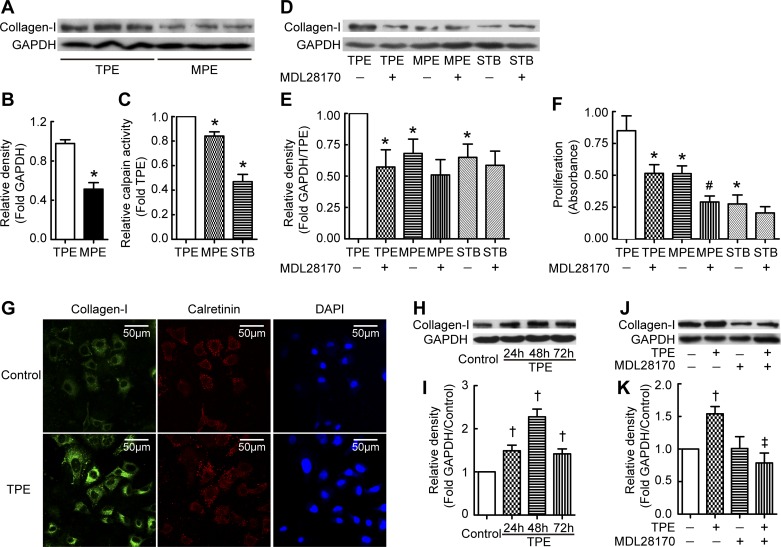
Tuberculous pleural effusion (TPE) induces collagen-I synthesis and proliferation of pleural mesothelial cells (PMCs) via calpain. *A–F*: PMCs were incubated with TPE, malignant pleural effusion (MPE), or serum from the same patient with TPE (STB) in the presence or absence of the calpain inhibitor MDL28170 (20 μM) for 24 h, after which calpain activity and intracellular collagen-I protein levels were measured as described in materials and methods. Cell proliferation was detected using a bromodeoxyuridine (BrdU) kit according to the manufacturer's instructions. *A* and *D*: representative immunoblots of collagen-I proteins. *B*: changes in relative density of collagen-I to GAPDH according to *A* (*n* = 8). *C*: relative calpain activity in PMCs (*n* = 3). *E*: changes in relative density of collagen-I to GAPDH according to *D* (*n* = 5). *F*: changes in cell proliferation of PMCs (*n* = 8). *G*: immunofluorescence staining of collagen-I in PMC. PMCs were incubated with or without TPE for 24 h and then stained using antibodies against collagen-I and calretinin. *H*: TPE induced time-dependent increases in collagen-I synthesis. PMCs were incubated with TPE for 24, 48, and 72 h, after which intracellular collagen-I protein levels were measured. *I*: changes in relative density of collagen-I to GAPDH according to *H* (*n* = 4). *J*: calpain inhibitor prevented TPE-induced collagen-I synthesis in primary PMCs. Primary PMCs were taken from rat pleura and then treated with TPE in the presence or absence of calpain inhibitor MDL28170 for 24 h, after which intracellular collagen-I protein levels were measured. *K*: changes in relative density of collagen-I to GAPDH according to *J* (*n* = 3). Data are expressed as mean ± SE. *P < 0.05 vs. TPE, #*P* < 0.05 vs. MPE, †*P* < 0.05 vs. control, ‡*P* < 0.05 vs. TPE.

To study the effects of TPE or MPE on collagen-I synthesis, PMCs were incubated with pleural fluid from patients with TPE or MPE for 24 h, and the protein levels of collagen-I were measured using Western blot analysis. The clinical characteristics of patients were disclosed in Table 1. As shown in Fig. 1, *A* and *B*, the protein levels of collagen-I were much higher in PMCs incubated with TPE than those with MPE.

**Table 1.** Clinical characteristics of patients with tuberculous pleural effusion and malignant pleural effusion

Characteristics	TPE	MPE	*P*
Gender	*n* = 56	*n* = 32	
Male	39	17	
Female	17	15	
Age, yr	43 (16–74)	60 (31–71)	<0.001
Pleural fluid			
ADA, U/l	41 (8–140)	12 (2–76)	<0.001
LDH, U/l	332 (119–1269)	269 (79–3373)	0.377
Protein, g/l	49.4 (25–59)	43.0 (36–53)	0.015
Total cell counts, ×10^9^/l	3.7 (1.0–13.3)	6.8 (0.2–310.0)	0.356
Lymphocytes, %	82 (20–97)	80 (20–95)	0.642

Data are presented as median (range). TPE, tuberculous pleural effusion; MPE, malignant pleural effusion; ADA, adenosine deaminase; LDH, lactate dehydrogenase. Comparisons were done by the Mann-Whitney U-test.

To investigate the mechanism of TPE-induced increase in collagen-I synthesis in PMCs, we detected calpain activity in PMCs treated with pleural fluid from patients with TPE or MPE. Calpain activity in PMCs incubated with pleural fluid from patients with TPE was significantly higher than that in PMCs incubated with pleural fluid from patients with MPE or with serum from the same patient with TPE (STB) (Fig. 1*C*). To further study the role of calpain in collagen-I synthesis, the calpain inhibitor MDL28170 was used to block calpain activity in PMCs. As shown in Fig. 1, *D* and *E*, MDL28170 prevented TPE-induced increase in collagen-I synthesis in PMCs. These results suggest that TPE induces collagen-I synthesis by activating calpain in PMCs.

To determine the mechanism of TPE-induced pleural fibrosis, proliferation of PMCs was measured. PMCs were incubated with pleural fluids from patients with TPE and MPE or with STB for 24 h, and cell proliferation was quantified by measuring BrdU incorporation. The increase in proliferation of PMCs incubated with pleural fluid from patients with TPE was much higher than that in cells incubated with MPE or STB (Fig. 1*F*). The calpain inhibitor MDL28170 dramatically reduced the TPE-induced increase in the proliferation of PMCs (Fig. 1*F*), suggesting that TPE-induced PMC proliferation is also through calpain.

To further confirm the effect of TPE and calpain on collagen-I synthesis in PMCs, we did immunofluorescence test and time course to show collagen-I expression. As shown in Fig. 1*G*, expression of collagen-I in TPE-treated PMCs was stronger than that in control. In the time-course investigation, TPE induced increases in collagen-I synthesis at 24, 48, and 72 h (Fig. 1, *H* and *I*). Moreover, in the primary rat PMCs, TPE also increased the protein levels of collagen-I, and this was blocked by calpain inhibitor, which was as same as found in Met-5A cells (Fig. 1, *J* and *K*).

#### The calpain inhibitor MDL28170 prevents TPE-induced collagen-I synthesis through inhibiting the PI3K/Akt/NF-κB signaling pathway.

**Fig. 2.**
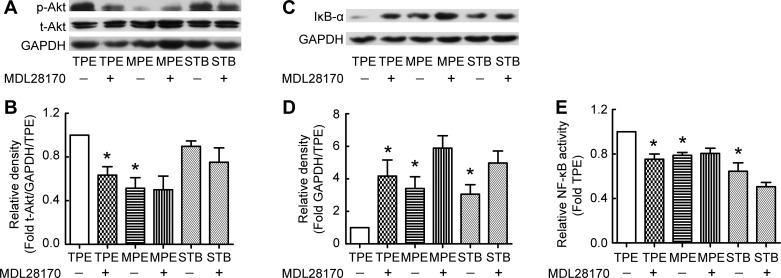
Calpain mediates TPE-induced collagen-I synthesis through the phosphatidylinositol 3-kinase (PI3K)/Akt/NF-κB signaling pathway. PMCs were incubated with TPE, MPE, or STB in the presence or absence of the calpain inhibitor MDL28170 (20 μM) for 24 h, after which protein levels of p-Akt, t-Akt, IκB-α, and collagen-I were measured using Western blots, and NF-κB activity was detected as described in materials and methods. *A* and *C*: representative immunoblots of p-Akt, t-Akt, and IκB-α. *B*: changes in relative density of p-Akt protein to t-Akt and GAPDH according to *A* (*n* = 5). *D*: changes in relative density of IκB-α protein to GAPDH according to *C* (*n* = 4). *E*: changes in relative NF-κB activity in PMCs (*n* = 4). Data are expressed as mean ± SE. *P < 0.05 vs. TPE.

The PI3K/Akt/NF-κB signaling pathway is important in cell proliferation and metabolism of the extracellular matrix. We studied the role of the PI3K/Akt/NF-κB signaling pathway in calpain-mediated collagen-I synthesis induced by TPE. As shown in Fig. 2, *A* and *B*, TPE induced the phosphorylation of Akt, and the calpain inhibitor MDL28170 prevented the TPE-induced increase in p-Akt. There was no significant change in t-Akt levels. The levels of IκB-α in PMCs treated with pleural fluid from patients with TPE were lower than those incubated with MPE and those incubated with STB. MDL28170 prevented IκB-α degradation induced by TPE in PMCs (Fig. 2, *C* and *D*). Correspondingly, NF-κB activities induced by TPE were much higher than those induced by MPE or STB, and MDL28170 inhibited NF-κB activation induced by TPE (Fig. 2*E*). These data suggest that the PI3K/Akt/NF-κB signaling pathway plays a critical role in calpain-mediated collagen-I synthesis induced by TPE.

#### Higher levels of ACE in TPE than that in MPE.

**Fig. 3.**
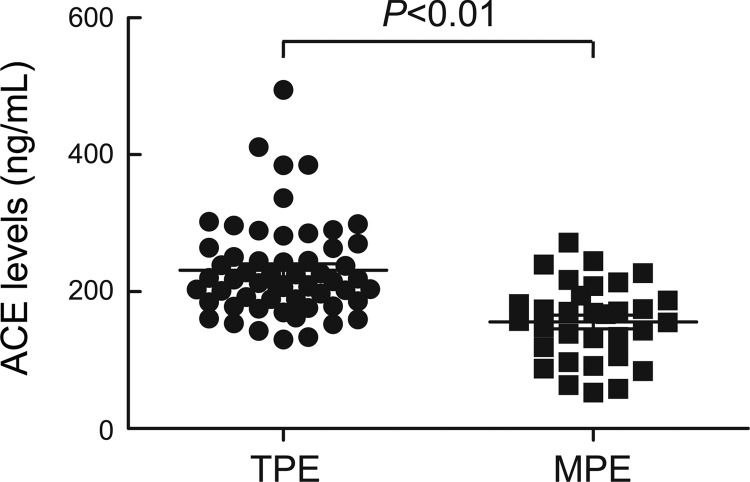
The levels of ACE in TPE are higher than that in MPE. ACE protein levels were measured in pleural fluids from patients with TPE (*n* = 56) and MPE (*n* = 32) using ELISA kit according to the manufacturer's instructions. Horizontal bars indicate means ± SE.

To study whether ACE is associated with pleural fibrosis induced by TPE, protein levels of ACE were assessed in pleural fluid from patients with TPE or MPE. As shown in Fig. 3, the protein levels of ACE in pleural fluid from patients with TPE were significantly higher than those with MPE.

#### ANG II induces collagen-I synthesis and proliferation of PMCs through calpain.

**Fig. 4.**
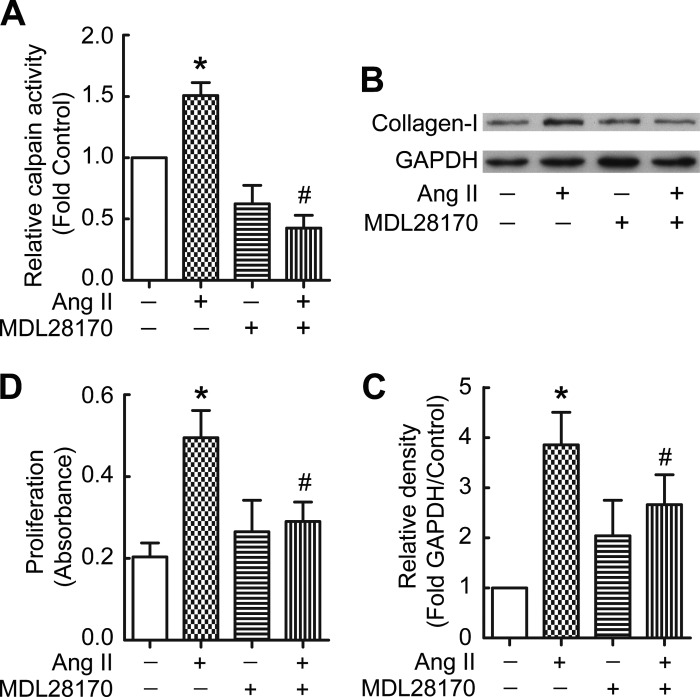
ANG II induces collagen-I synthesis and cell proliferation via activation of calpain in PMCs. PMCs were incubated with ANG II (10^−7^ M) in the presence or absence of the calpain inhibitor MDL28170 (20 μM) for 24 h, after which calpain activity was detected as described in Methods, intracellular collagen-I protein levels were measured by Western blots, and cell proliferation was detected by BrdU kit. *A*: relative calpain activity in PMCs (*n* = 3). *B*: representative immunoblots of collagen-I. *C*: changes in relative density of collagen-I protein to GAPDH according to *B* (*n* = 3). *D*: changes in cell proliferation of PMCs (*n* = 8). Data are expressed as mean ± SE. *P < 0.05 vs. control, #*P* < 0.05 vs. ANG II.

To further confirm the role of ACE in TPE-induced collagen-I synthesis and proliferation of PMCs, PMCs were incubated with ANG II in the absence or presence of the calpain inhibitor MDL28170. As shown in Fig. 4, ANG II induced an increase in calpain activity (Fig. 4*A*) and collagen-I synthesis (Fig. 4, *B* and *C*) in PMCs, and in PMC proliferation (Fig. 4*D*). Moreover, MDL28170 prevented ANG II-induced collagen I synthesis and proliferation of PMCs (Fig. 4, *B–D*). These data suggest that ANG II recapitulates the effect of TPE on PMCs and that ANG II-induced collagen-I synthesis and proliferation of PMCs are through calpain.

#### Calpain-mediated PI3K/Akt/NF-κB signaling pathway is involved in ANG II-induced collagen-I synthesis and cell proliferation.

**Fig. 5.**
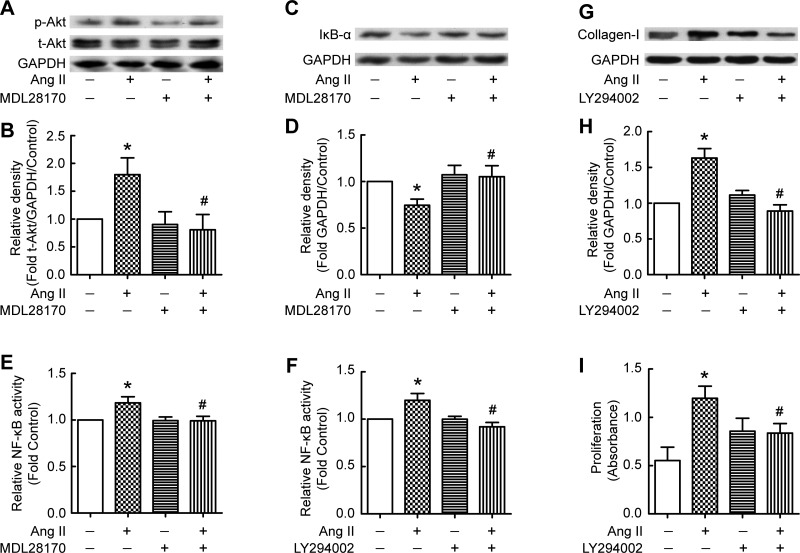
Calpain mediates ANG II-induced collagen-I synthesis through the PI3K/Akt/NF-κB signaling pathway. PMCs were incubated with ANG II (10^−7^ M) in the presence or absence of the calpain inhibitor MDL28170 (20 μM) or the PI3K inhibitor LY294002 (20 μM) for 24 h, after which intracellular p-Akt, t-Akt, IκB-α, and collagen-I protein contents were measured, and NF-κB activity was detected. *A*, *C*, and *G*: representative immunoblots of p-Akt, t-Akt, IκB-α, collagen-I, and GAPDH proteins in PMCs. *B*: changes in relative density of p-Akt to t-Akt and GAPDH according to *A* (*n* = 6). *D*: changes in relative density of IκB-α to GAPDH according to *C* (*n* = 4). *E*: changes in relative NF-κB activity in PMCs treated with ANG II in the presence or absence of MDL28170 (*n* = 8). *F*: changes in relative NF-κB activity in PMCs treated with ANG II in the presence or absence of LY294002 (*n* = 6). *H*: changes in relative density of collagen-I to GAPDH according to *G* (*n* = 5). *I*: changes in cell proliferation (*n* = 8). Data are expressed as mean ± SE. *P < 0.05 vs. control, #*P* < 0.05 vs. ANG II.

We studied the role of the PI3K/Akt/NF-κB signaling pathway in calpain-mediated collagen-I synthesis induced by ANG II. As shown in Fig. 5, *A* and *B*, ANG II induced phosphorylation of Akt, and the calpain inhibitor MDL28170 blocked the ANG II-induced increase in p-Akt. ANG II induced degradation of IκB-α, which was prevented by MDL28170 (Fig. 5, *C* and *D*). Furthermore, the PI3K inhibitor LY2940002 prevented the ANG II-induced collagen-I synthesis and proliferation of PMCs (Fig. 5, *G–I*). Both the calpain inhibitor MDL28170 and the PI3K inhibitor LY2940002 prevented ANG II-induced increases in NF-κB activity (Fig. 5, *E* and *F*). These data suggest that ANG II-induced collagen I synthesis and cell proliferation in PMCs were also through the calpain-mediated PI3K/Akt/NF-κB signaling pathway and that the ANG II might be related to TPE-induced collagen-I synthesis and cell proliferation in PMCs.

#### ANG II type 1 receptor participates in TPE-induced collagen-I synthesis in PMCs.

**Fig. 6.**
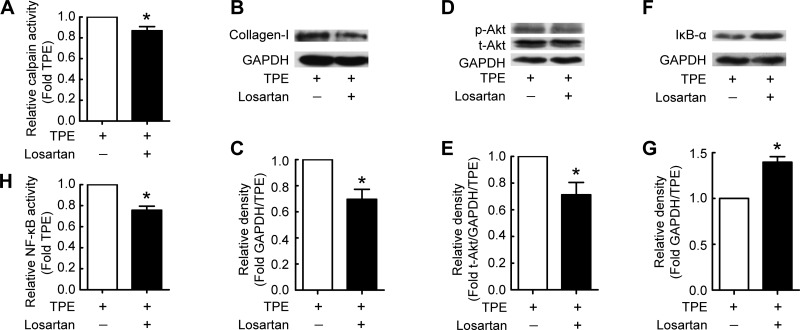
TPE induces collagen-I synthesis via ANG II type 1 receptor (AT_1_R) in PMCs. PMCs were incubated with TPE in the presence or absence of AT_1_R antagonist (losartan) for 24 h, after which calpain and NF-κB activity were detected as described in materials and methods, and intracellular p-Akt, t-Akt, and IκB-α protein contents were measured by Western blots. *A*: changes in calpain activity in PMCs (*n* = 4). *B*, *D*, and *F*: representative immunoblots of collagen-I, p-Akt, t-Akt, and IκB-α proteins in PMCs. *C*: changes in relative density of collagen-I protein to GAPDH according to *B* (*n* = 5). *E*: changes in relative density of p-Akt protein to t-Akt and GAPDH according to *D* (*n* = 5). *G*: changes in relative density of IκB-α protein to GAPDH according to *F* (*n* = 5). *H*: changes in relative NF-κB activity (*n* = 4). Data are expressed as mean ± SE. *P < 0.05 vs. TPE.

To further investigate the role of ANG II in TPE-induced collagen-I synthesis and cell proliferation, PMCs were incubated with pleural fluid from patients with TPE in the absence or presence of the ANG II type 1 receptor (AT_1_R) antagonist losartan. We found that losartan inhibited the TPE-induced increase of calpain activity (Fig. 6*A*) and collagen-I synthesis in PMCs (Fig. 6, *B* and *C*). Losartan also decreased the p-Akt level (Fig. 6, *D* and *E*). The degradation of IκB-α and the increase in NF-κB activity induced by TPE were inhibited in PMCs in the presence of losartan (Fig. 6, *F–H*). These results give further support that ANG II is responsible for TPE-induced collagen I synthesis and cell proliferation in PMCs.

#### Activation of calpain and collagen deposition in the pleural samples from patients with tuberculous pleurisy.

**Fig. 7.**
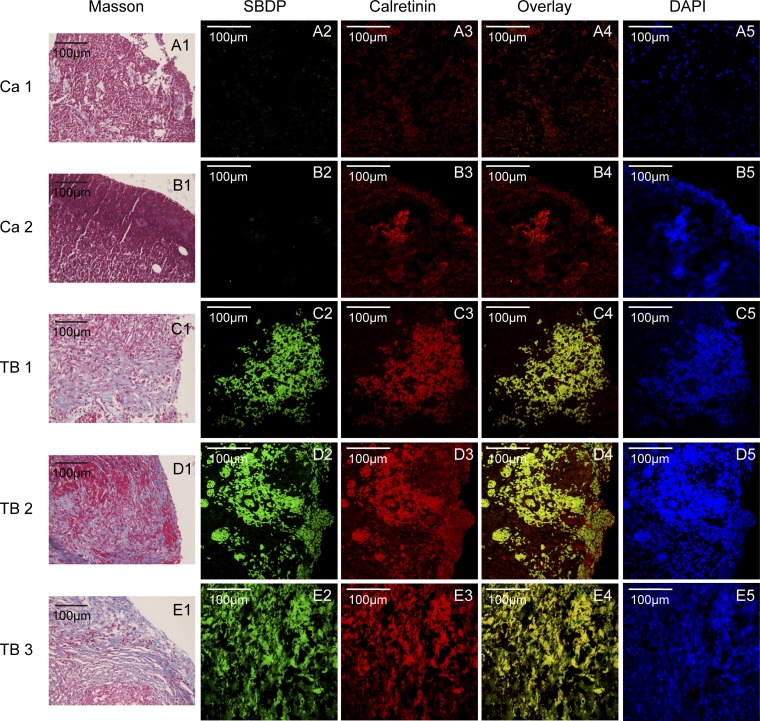
Activation of calpain and collagen deposition in the pleural samples from patients with tuberculous pleurisy. Pleural tissue samples were collected from parietal pleura in patients with TPE (TB 1, 2, 3) or MPE (Ca 1, 2). The pleural sections were stained with Masson staining for morphological analysis. spectrin breakdown product (SBDP) and calretinin (marker for PMCs) were stained by immuofluorescence staining. *A1–E1*: Masson stainings, blue color shows collagen. *A2–E2*: immuofluorescence stainings of SBDP (green color). *A3–E3*: immuofluorescence stainings of calretinin (red color). *A4–E4*: overlays of immuofluorescence stainings, yellow color shows colocalization of SBDP and calretinin. *A5–E5*: DAPI stainings.

To address the clinical relevance of these observations in cells and the animal model, we determined the levels of calpain activation by measuring protein levels of the specific calpain cleavage product SBDP. As shown in Fig. 7, there were massive deposits of collagen in the pleural sections from patients with tuberculous pleurisy. Furthermore, the pleural sections from patients with tuberculous pleurisy contained a very high level of SBDP that was colocalized with calretinin, a marker of PMCs, as shown in the overlay images. Together, these results indicate that calpain activity is markedly increased in PMCs of patients with tuberculous pleurisy that is complicated with pleural fibrosis.

## DISCUSSION

We show here for the first time that calpain plays an important role in tuberculous pleural fibrosis. We found that TPE induces calpain activation in PMCs and that inhibition of calpain prevents TPE-induced collagen-I synthesis and cell proliferation of PMCs. Moreover, this study revealed that activation of calpain and subsequent triggering of the PI3K/Akt/NF-κB signaling pathway in PMCs result from ACE-ANG II system in TPE. At last, calpain activation and collagen deposition are confirmed in the pleural biopsy samples from patients with tuberculous pleurisy. These observations indicate that activation of calpain by ANG II mediates TPE-induced collagen-I synthesis and pleural fibrosis.

ANG II generated locally in tissues has been shown to be a key mediator in the pathogenesis of tissue remodeling (9, 14, 17, 29, 30). ACE and ANG II are involved in fibrotic diseases, such as cardiovascular fibrosis (30) and pulmonary fibrosis (29). ANG II has been reported to activate calpain in vascular smooth muscle cells (24). In the present study, we found that the protein levels of ACE are significantly higher in pleural fluid of patients with TPE than those with MPE. ANG II recapitulates the TPE-induced activation of calpain and the PI3K/Akt/NF-κB signaling pathway. Notably, our data showed that inhibition of calpain prevents ANG II-induced increases in collagen synthesis and cell proliferation of PMCs. In addition, blocking the ANG II receptor AT_1_R using losartan inhibits TPE-induced increase in calpain activity, Akt phosphorylation, NF-κB activation, and collagen-I synthesis in PMCs. These results indicate that the activation of calpain and subsequent triggering of the PI3K/Akt/NF-κB signaling pathway in PMCs contribute to the ACE-ANG II system in TPE.

Collagen synthesis is an important function of PMCs that participate in pleural fibrosis. Disordered fibrin turnover plays an important role in the pathogenesis of pleural fibrosis. With ongoing remodeling rather than clearance of transitional fibrin, collagen deposition occurs, which ultimately leads to progressive scarring and fibrotic repair (23). The subpleural fibroblasts are conventionally considered to be the primary target cells for pleural fibrosis. Nevertheless, increasing evidence indicates that PMCs play a significant role in the pathogenesis of pleural fibrosis. PMCs produce large amounts of extracellular matrix components including collagen (16, 23). After the epithelial to mesenchymal transition, PMCs have an increased capacity for collagen synthesis, resulting in overdeposition of collagen (18, 35). TGF-β_1_ is a classical fibrotic factor and standard control in fibrosis investigation. To confirm the biological activity of MeT-5A (line cells of PMC), we have treated them with TGF-β_1_ and found TGF-β_1_ induced increases in collagen-I synthesis (7). In the current study, we still used Met-5A cells as cell model. We found PMCs stimulated with TPE and ANG II produce large amounts of collagen and undergo hyperproliferation. The calpain inhibitor MDL28170 prevents TPE- and ANG II-induced collagen-I synthesis and cell proliferation. These findings suggest that PMCs represent an important source of excessive collagen production induced by TPE and that calpain is a key mediator in this process.

As a typical cell proliferation and extracellular matrix regulation signaling pathway, the PI3K/Akt/NF-κB pathway has been reported to be involved in regeneration of PMCs (31). In the present study, we provided evidence that calpain mediates collagen synthesis and pleural fibrosis induced by TPE through the PI3K/Akt/NF-κB signaling pathway. Previous studies using calpain knockout cells have shown that activation of the PI3K/Akt signaling downstream of multiple signaling pathways is dependent on calpain (27). There are several possible mechanisms for calpain-mediated activation of PI3K/Akt/NF-κB signaling pathway. First, calpain exerts its function by limited cleavage of its substrates. Several calpain substrates, including the phosphatase and tensin homolog on chromosome 10 (PTEN) and protein phosphatase 2A (PP2A), have been shown to inhibit the activation of Akt (4, 6). PTEN inhibits activation of Akt by converting PIP_3_ into PIP_2_, and reduction of the level of PTEN causes the activation of Akt in pulmonary fibrosis (33, 34). Degradation of PTEN and PP2A by calpain may result in an increase in p-Akt and Akt activation (2). Second, calpain can directly degrade IκB and induce NF-κB activation (13). We found that inhibition of calpain using MDL28170 reduces the decrease in IκB levels and the increase in NF-κB activity induced by TPE, suggesting that calpain may directly activate NF-κB. Third, p-Akt induces the phosphorylation of IκB and promotes IκB dissociation from NF-κB, leading to NF-κB activation (21). Consistently, we found that inhibition of PI3K/Akt signaling prevented ANG II-induced increases in NF-κB activity, suggesting that NF-κB activation may also be caused by the calpain-promoted Akt activation.

Finally, we determined the level of calpain activation by measuring levels of SBDP in the pleural tissues of tuberculous and malignant pleurisy patients. We observed increased calpain activity in PMCs of tuberculous pleurisy patients compared with malignant pleurisy patients. Calretinin is a marker of mesothelial cells. The samples were taken from the pleura, so the calretinin-positive cells are PMCs. Just as we did in the cell models, PMCs treated with TPE were producing increased collagen-I. As a special epithelial cell, mesothelial cell can undergo epithelial to mesenchymal transition (18, 35). Therefore, in the process of pleural fibrosis, PMCs also can undergo differentiation into mesenchymal cells and produce more collagen-I. These findings provide evidence that calpain in PMCs might be a novel target for intervention in tuberculous pleural fibrosis.

In summary, our findings support that ANG II-activated calpain mediates TPE-induced collagen-I synthesis and proliferation of PMCs via the PI3K/Akt/NF-κB signaling pathway. More importantly, calpain plays an important role in tuberculous pleural fibrosis and might be a novel target for intervention in the disease.

## GRANTS

This work was supported in part by grants from National Natural Science Foundation of China Grants 81370186, 81573485, and 31271490 (to W. L. Ma); 81300047 (to F. Xiang); 81570087 (to H. Ye); 81200020 (to P. C. Cai); 81470274 (to Q. Zhou); and 81470257 (to J. B. Xin) and Canadian Institute of Health Research (CIHR) (FRN81189 to P. A. Greer).

## DISCLOSURES

No conflicts of interest, financial or otherwise are declared by the author(s).

## AUTHOR CONTRIBUTIONS

J.Y., P.-C.C., H.Y., Y.S., and W.-L.M. conception and design of research; J.Y., F.X., P.-C.C., Y.-Z.L., X.-X.X., F.-Z.L., and H.Y. performed experiments; J.Y., F.X., Y.-Z.L., F.Y., J.-B.X., and H.Y. analyzed data; J.Y., H.Y., and W.-L.M. prepared figures; F.X., P.A.G., H.-Z.S., Q.Z., and J.-B.X. interpreted results of experiments; Y.S. and W.-L.M. drafted manuscript; Y.S. and W.-L.M. edited and revised manuscript; Y.S. and W.-L.M. approved final version of manuscript.
